# Knowledge mapping of spastic cerebral palsy. A bibliometric analysis of global research (2000–2022)

**DOI:** 10.1186/s13052-024-01577-1

**Published:** 2024-01-18

**Authors:** Xing Wang, Siew Hoon Teh, Xing-hua Wang

**Affiliations:** 1 M. Kandiah Faculty of Medicine and Health Sciences, Universiti Tunku Abdul Rahman, Kajang, Selangor Malaysia; 2 Famous TCM Expert Heritage Studio, Xi’an Encephalopathy Hospital Affiliated to Shaanxi University of Chinese Medicine, Xi’an, Shaanxi, China

**Keywords:** Spastic cerebral palsy, Bibliometric analysis, Knowledge structure, Hotspots

## Abstract

**Background:**

Cerebral palsy (CP) is characterized by abnormal pronunciation, posture, and movement. Spastic CP accounts for more than 70% of all CP. To date, there has been no bibliometric analysis to summarize study on spastic CP. Here, we aim to conduct a bibliometric analysis of spastic CP to summarize this field's knowledge structure, research hotspots, and frontiers.

**Method:**

Publications about spastic CP were searched utilizing the Web of Science Core Collection (WoSCC) database from 1 January 2000 to 30 November 2022, the WoSCC literature analysis wire, VOSviewer 1.6.18, CiteSpace 6.1.R4 and Online analysis platform for bibliometrics were used to conduct the analysis.

**Results:**

A total of 3988 publications, consisting of 3699 articles and 289 reviews, were included in our study. The United States emerged as the most productive country, while Kathleen Univ Leuven was the most productive institution. The leading author was Desloovere K. A total of 238 journals contributed to this field, with Developmental medicine and child neurology being the leading journal. Important keywords and keyword clusters included Spastic cerebral palsy, Reliability, and Gross motor function. Keywords identified through burst detection indicated that hotspots in this field were management, randomized controlled trials, and definition.

**Conclusion:**

Based on the analysis of bibliometric on spastic CP over the past 20 years, the trends and the knowledge graph of the countries, institutions, authors, references, and the keywords have been identified, providing accurate and expedited insights into critical information and potentially new directions in the study of spastic CP.

## Background

Cerebral palsy (CP) is characterized by abnormal pronunciation, posture and movement. Clinically, it is divided into spastic hemiplegia, diplegia, quadriplegia, involuntary movement, ataxia, and mixed type, according to the main motor syndrome [1, 2]. Spastic CP accounts for more than 70% of all CP [3, 4]. At present, the etiology of CP is not clear. Studies have shown that complications such as low birth weight, birth asphyxia, and placental and abnormal fetal position are closely related [5]. Several population-based research reports around the world show that the prevalence of the disease is estimated to be about 1.5–4 per 1000 children [6–10], and the prevalence of CP in China aged 0–18 and different geographical regions is 1.66–2.47 ‰, which is increasing year by year [11]. The children's poor social adaptability caused by abnormal motor function and intellectual impairment has brought a tremendous mental burden to them and their families. At the same time, the long-term need for medical services makes the children's families and society have to bear enormous economic pressure [12].

Nowadays, spastic CP has become a hot topic for many scholars around the world. More and more new methods, such as botulinum toxin A injection [13, 14], stem cell transplantation [15, 16], and Traditional Chinese Medicine [17, 18], have been used for the treatment of this disease, all with good results. However, there is no literature to assess the published related literature systematically. Bibliometric analysis can more objectively and comprehensively describe a particular field's historical overview, research focus, and development trend [19–21]. Some biometric tools have been applied to scientific metrology, including CiteSpace [22], CitNetExplorer [23], VOSviewer [24], and HistCite [25], to assess the overview of academic fields. In this paper, Bibliometric analysis software or website were used to evaluate the bibliographic catalogs in spastic CP research. The objectives of this study include (1) summarizing the historical features of the spastic CP literature; (2) recognizing the hotspots of the research field; (3) revealing study trends for future research.

## Materials and methods

The WoSCC has been widely used for bibliometric analysis widely [26, 27], which has the largest authoritative, comprehensive, and systematic academic information resource covering the top number of disciplines in the world [28]. In our study, publications about spastic CP were taken from the WoSCC. The search term used were TS = “Cerebral palsy” and “Spastic”. The search results were narrowed by publication data, including a date range of 1 January 2000 to 30 November 2022, a publication language of English, and the article types of reviews and articles.

Excel 2019(Microsoft, Redmond, Washington, USA) was used to analyze the annual growth of publication trends and major contributors (e.g., authors, institutions, countries, and journals). The WoSCC literature analysis wire, VOSviewer(version 1.6.18, Leiden University, Leiden, Netherlands) and CiteSpace (Version 6.1.R4) and Online analysis platform for bibliometrics were used to construct the knowledge network, identify the collaborative network, the keyword co-occurrence network, and the co-citation references network. Total link strength (TLS) was used to evaluate the cooperation relationship. The node size was positively related.

## Results

### General data

**Fig. 1:**
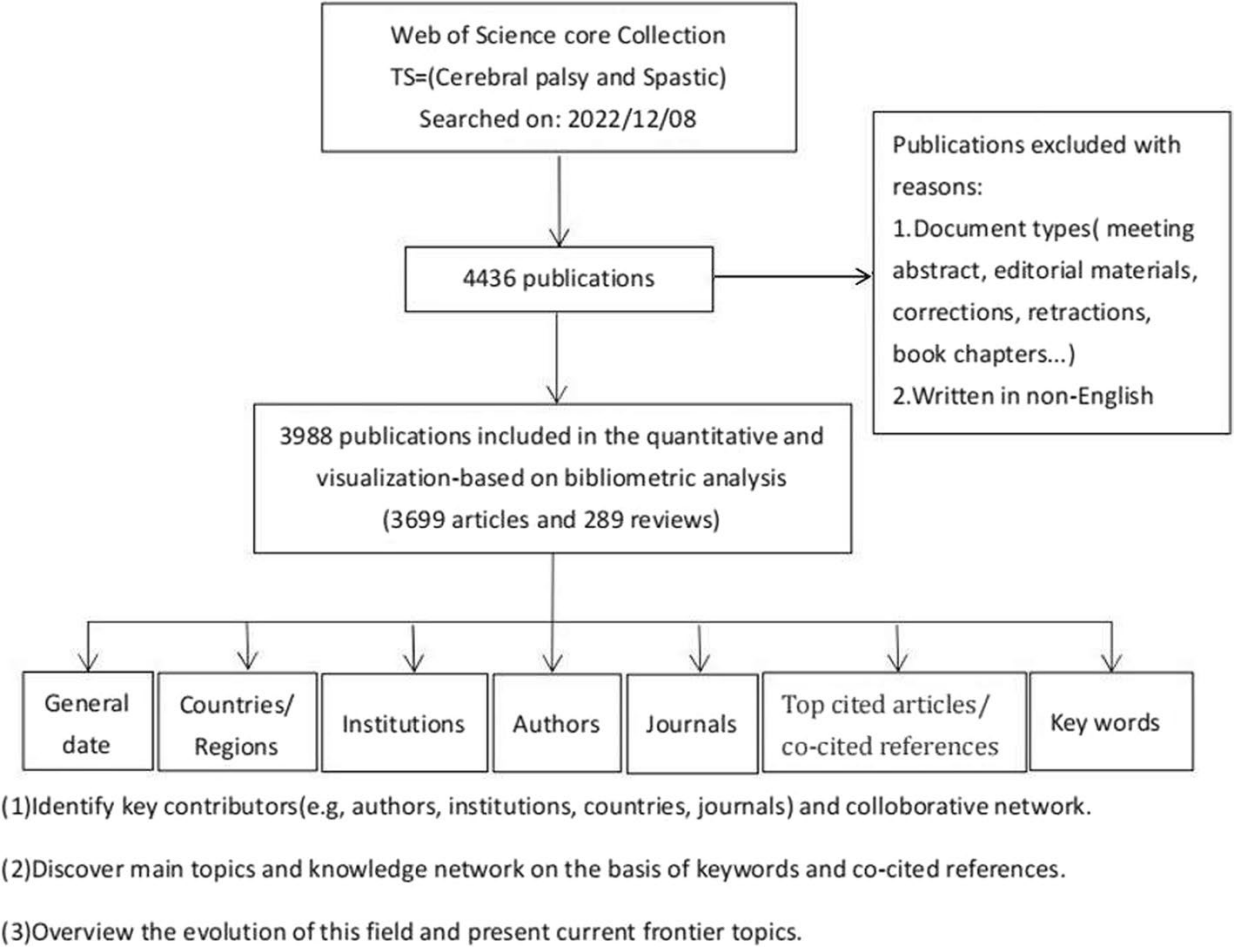
The workflow of data collection and bibliometric analysis

Figure 1 shows the process of literature searching and bibliometric analysis. A total of 3988 publications were retrieved and imported into VOSviewer, CiteSpace, and the Online analysis platform for further analysis. The total citations (TC) total was 92,603, and the number of citations per publication (CPP) was 23.22.

### Publication trend

**Fig. 2:**
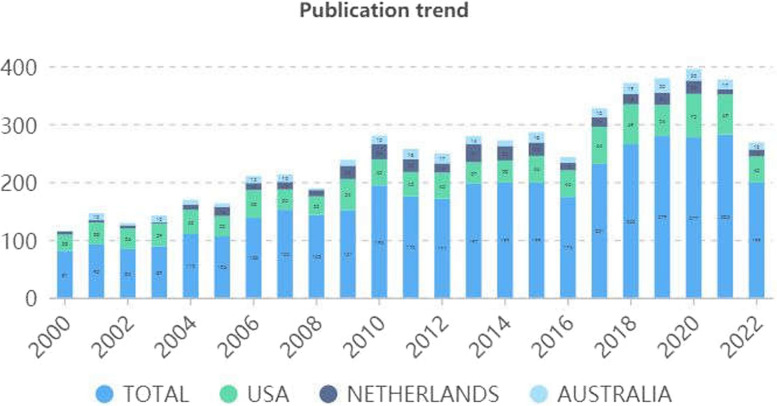
Publication trend of the top three prolific countries/regions

The number of annual publications in the spastic CP field exhibited an upward trend and could be roughly divided into three phases. Phase I was from 2000 to 2005, with below 150 publications per year. Phrase II was from 2006 to 2015, and the number of publications steadily increased from 150 to over 250 per year. Phase III was from 2015 to 2022, the number slightly decreased in 2015 and 2016 and increased sharply after 2016 to over 300 publications per year. Figure 2 presents the annual output of the top three contributing countries.

### Country/region and Institution contributions

**Fig. 3:**
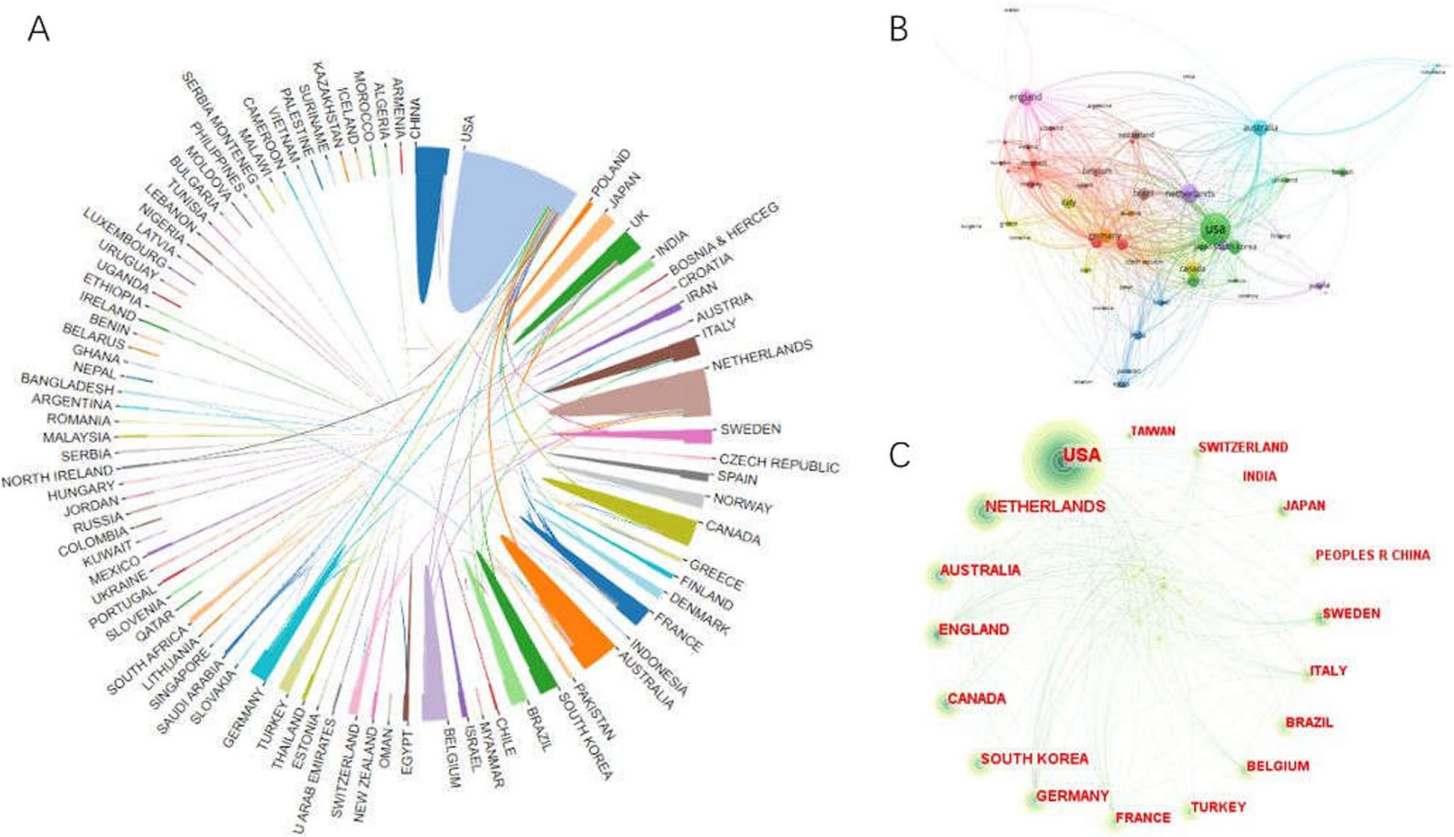
**A** The collaboration network of Countries/Regions generated by the Online analysis platform for bibliometrics. **B** The collaboration network of Countries/Regions generated by VOSviewer. **C** The collaboration network of Countries/Regions generated by Citespace

Overall, a total of 3,487 institutions and 94 countries/regions contributed to this field. As depicted in Table 1, The USA contributed the most papers in this field (1062 publications Total citation (TC)(31,220), Citations per publication (CPP)(28.788)), followed by Netherlands (356 publications, TC(9467), CPP(26.62)), Australia (290 publications, TC(9907), CPP(34.16)), implying that the contributions of the three Countries were significantly more than that of the other countries in this field, followed by England (268 publications), Canada (223 publications), South Korea (211 publications). Figure 3 shows the collaborative network between countries. The country/region with the highest total link strength (TLS) was the USA (520), followed by England (317), which exhibited a close mutual cooperative relationship. South Korea has high papers (211) but low TLS (45).

**Table 1:** Top 10 most productive Countries/Regions

	Countries/Regions	Record Count	Centrality	Total citation (TC)	Citations per publication (CPP)	Total link strength (TLS)
1	USA	1062	0.12	31,220	28,788	520
2	Netherlands	356	0.06	9467	26.62	269
3	Australia	290	0.00	9907	34.16	228
4	England	268	0.12	8740	32.61	317
5	Canada	223	0.04	6713	30.1	185
6	South Korea	211	0.00	3393	16.08	45
7	Germany	195	0.32	5652	28.98	253
8	France	168	0.04	3684	21.93	236
9	Turkey	166	0.04	2016	12.14	78
10	Belgium	165	0.21	4019	24.36	225

**Fig. 4:**
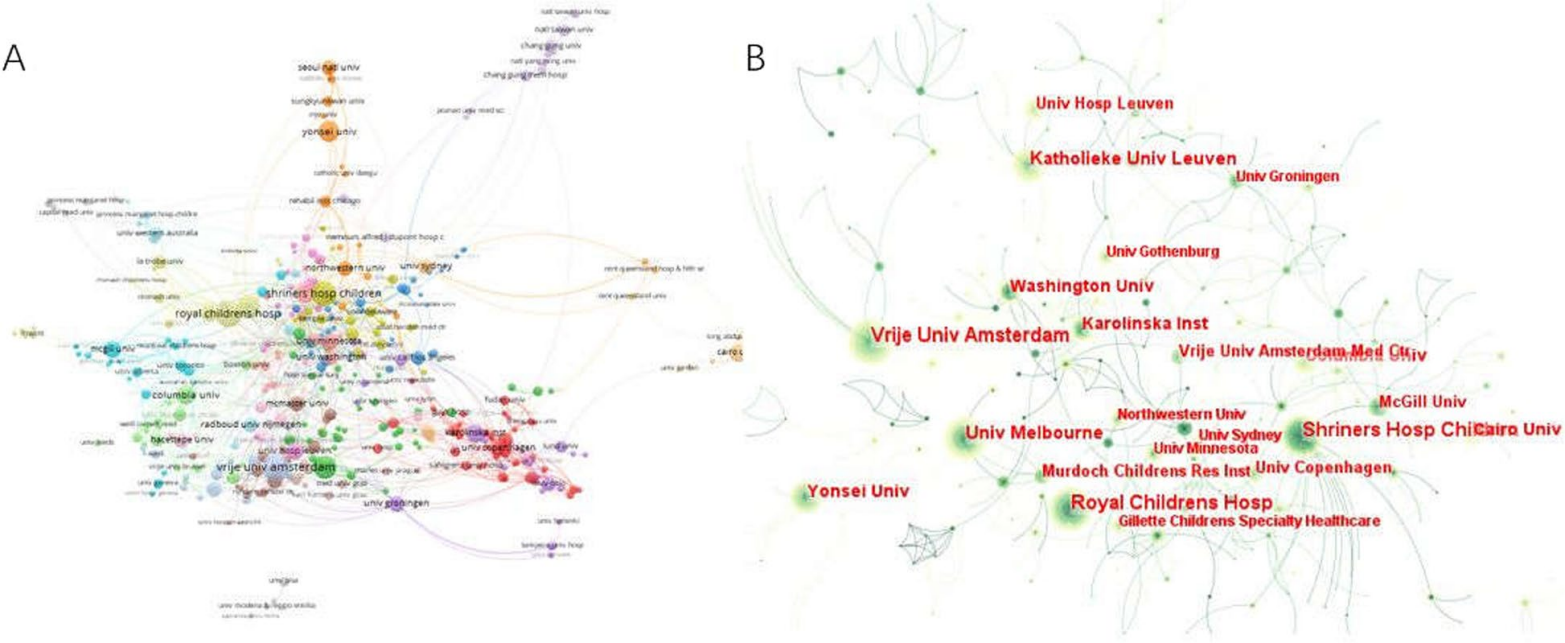
**A** The collaboration network of Institutions generated by VOSviewer. **B** The collaboration network of Institutions generated by Citespace

The institution with the most publications related to spastic CP research is listed in Table 2. The Kathleen Univ Leuven, with the most publications (177), TC (1421), and CPP (8.03), preceded the Vrije Univ Amsterdam and the Yonsei Univ. Additionally, in terms of CPP, the Royal Children's Hosp was at the top (16.04), with McGill Univ (11.83) and the Univ Melbourne (11.01) following closely. Figure 4 shows weak collaborative relationships among institutions, suggesting the need for greater collaboration. The institution with the highest TLS was the Royal Children's Hosp (193), followed by Univ Melbourne (171).

**Table 2:** Top 10 most productive institutions

	Institutions	Record Count	Centrality	Total citation (TC)	Citations per publication (CPP)	Total link strength (TLS)
1	Kathleen Univ Leuven	177	0.05	1421	8.03	129
2	Vrije Univ Amsterdam	177	0.11	1213	6.85	169
3	Yonsei Univ	140	0.05	761	5.44	25
4	Royal Children’s Hosp	138	0.08	2213	16.04	193
5	Shriners Hosp Children	127	0.13	1131	8.91	102
6	McGill Univ	121	0.05	1431	11.83	43
7	Washington Univ	107	0.18	694	6.49	73
8	Univ Melbourne	91	0.09	1002	11.01	171
9	Univ Hosp Leuven	82	0.01	400	4.88	84
10	Vrije Univ Amsterdam Med Ctr	74	0.05	698	9.43	55

### Author contributions

**Fig. 5:**
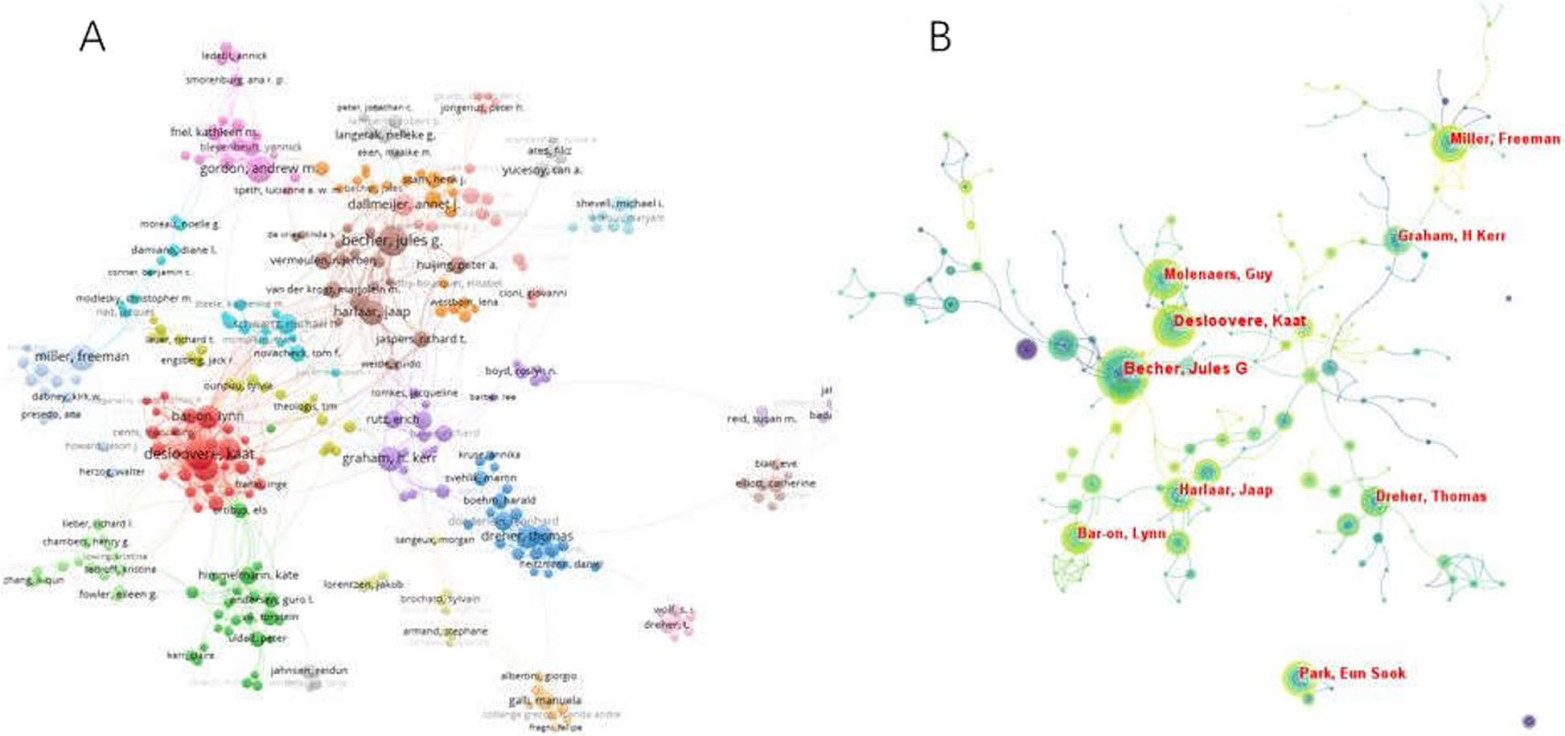
**A** The collaboration network of Authors generated by VOSviewer. **B** The collaboration network of Authors generated by Citespace

The top 10 authors contributed a total of 523 publications in this field. Desloovere K with the most publications (65), TC (1309), and CPP (20.14), followed by Becher JG with publications (64), TC (1638), and CPP (25.59). Graham HK, with publications (64), has the highest TC (4042) and CPP (63.16) (Table 3). As shown in Fig 5, the top 5 authors with the largest TLS were as follows: Desloovere K (256), Molenaers G (180), Becher JG (179), Dreher T (129), and Harlaar J (104). The connections between authors from different countries are inadequate.

**Table 3:** Top 10 most productive authors

	Authors	Record Count	Centrality	Total citation (TC)	Citations per publication (CPP)	Total link strength (TLS)
1	Desloovere K	65	0.01	1309	20.14	256
2	Becher JG	64	0.00	1638	25.59	179
3	Graham HK	64	0.01	4042	63.16	60
4	Miller F	57	0.00	1118	19.61	91
5	Molenaers G	51	0.00	1453	28.49	180
6	Dreher T	49	0.00	840	17.14	104
7	Gordon AM	45	0.00	913	20.29	101
8	Park ES	44	0.00	905	20.57	47
9	Wolf SI	43	0.00	684	15.91	85
10	Harlaar J	41	0.02	805	19.63	129

### Journals contributions

Table 4 lists the top 10 productive journals involved in this study. Developmental Medicine and child neurology was the most productive journal with 418 publications in the spastic CP field, TC (5099), CPP (12.20), followed by Gait & Posture (196 publications), TC (1779), CPP (9.08), Journal of Pediatric Orthopaedics (137), TC (1254), CPP (9.15). Research in developmental disabilities (90), TC (502), CPP (5.58).

**Table 4:** Top 10 most productive journals

	Journals	Record Count	Centrality	Total citation (TC)	Citations per publication (CPP)	Total link strength (TLS)
1	DEVELOPMENTAL MEDICINE AND CHILD NEUROLOGY	418	0.95	5099	12.20	529,402
2	GAIT & POSTURE	196	0.03	1779	9.08	150,271
3	JOURNAL OF PEDIATRIC ORTHOPAEDICS	137	0.60	1254	9.15	165,401
4	RESEARCH IN DEVELOPMENTAL DISABILITIES	90	0.00	502	5.58	42,446
5	ARCHIVES OF PHYSICAL MEDICINE AND REHABILITATION	86	1.02	852	9.91	131,034
6	JOURNAL OF CHILD NEUROLOGY	71	0.03	329	4.63	49,891
7	JOURNAL OF PEDIATRIC ORTHOPAEDICS-PART B	70	0.00	433	6.19	25,025
8	DISABILITY AND REHABILITATION	67	0.10	405	6.04	53,608
9	CLINICAL BIOMECHANICS	55	0.03	378	6.87	34,570
10	PEDIATRIC PHYSICAL THERAPY	51	0.07	127	2.49	40,300

**Fig. 6:**
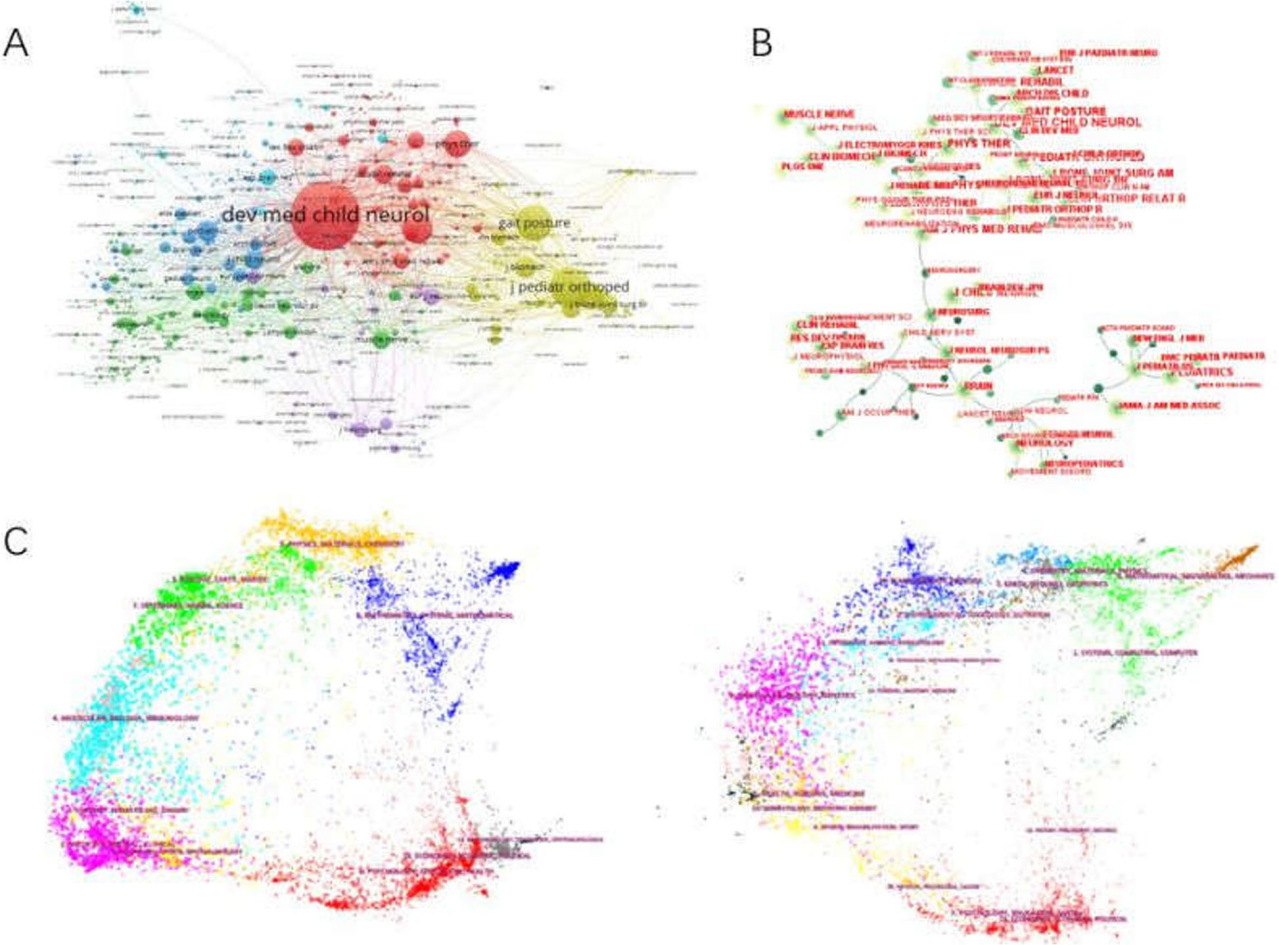
**A** The co-citation of journals generated by VOSviewer. **B** The co-citation of journals generated by Citespace. **C** The dual-map overlay of articles cited. (The left side was the citing journal, and the right side was the cited journal)

The journals of co-citation analysis are shown in Fig 6 The top 5 journals with TLS were as follows: Developmental medicine and child neurology (529,402), Journal of pediatric orthopaedics (165,401), Gait & Posture (150,271), Archives of physical medicine and rehabilitation (131,034), and Disability and rehabilitation (53,608).

### Analysis of high-cited references

The characteristics of publications are summarized in Table 5. The most-cited reference, "The epidemiology of cerebral palsy: Incidence, impairments and risk factors," was published in the DISABILITY AND REHABILITATION and authored by Odding, E in 2006. The second most-cited reference, “Classification and definition of disorders causing hypertonia in childhood," was published in PEDIATRICS and authored by Sanger, TD, in 2003. The third most-cited reference, "Use of a Low-Cost, Commercially Available Gaming Console (Wii) for Rehabilitation of an Adolescent with Cerebral Palsy," was published in the PHYSICAL THERAPY and authored by Deutsch in 2008. The top three most highly cited articles discussed the epidemic characteristics of CP [29], the definition and characteristics of hypertonia [30], and the low-cost rehabilitation game machine for cerebral [31]. It could be noted that the top ten highly cited articles were all published before 2010, which shows that there are not many breakthroughs in the research of spastic CP in this decade.

**Table 5:** Top 10 highest cited references

	Title	Source Title	Total Citations (TC)	Publication Date	First Author
1	The epidemiology of cerebral palsy: Incidence, impairments and risk factors	DISABILITY AND REHABILITATION	569	2006	Odding, E
2	Classification and definition of disorders causing hypertonia in childhood	PEDIATRICS	443	2003	Sanger, TD
3	Use of a Low-Cost, Commercially Available Gaming Console (Wii) for Rehabilitation of an Adolescent with Cerebral Palsy	PHYSICAL THERAPY	374	2008	Deutsch
4	Chorioamnionitis and cerebral palsy in term and near-term infants	JAMA-JOURNAL OF THE AMERICAN MEDICAL ASSOCIATION	369	2003	Wu, YW
5	Changing panorama of cerebral palsy in Sweden. VIII. Prevalence and origin in the birth year period 1991–94	ACTA PAEDIATRICA	362	2001	Hagberg, B
6	Spastic movement disorder: impaired reflex function and altered muscle mechanics	LANCET NEUROLOGY	349	2007	Dietz, Volker
7	Models of white matter injury: Comparison of infectious, hypoxic-ischemic, and excitotoxic insults	MENTAL RETARDATION AND DEVELOPMENTAL DISABILITIES RESEARCH REVIEWS	341	2002	Hagberg, H
8	Neurobiology of hypoxic-ischemic injury in the developing brain	PEDIATRIC RESEARCH	339	2001	Johnston, MV
9	Practice parameter: Diagnostic assessment of the child with cerebral palsy—Report of the Quality Standards Subcommittee of the American Academy of Neurology and the Practice Committee of the Child Neurology Society	NEUROLOGY	291	2004	Ashwal, S
10	The changing panorama of cerebral palsy in Sweden. IX. Prevalence and origin in the birth-year period 1995–1998	ACTA PAEDIATRICA	287	2005	Himmelmann, K

### Analysis of keywords

**Fig. 7:**
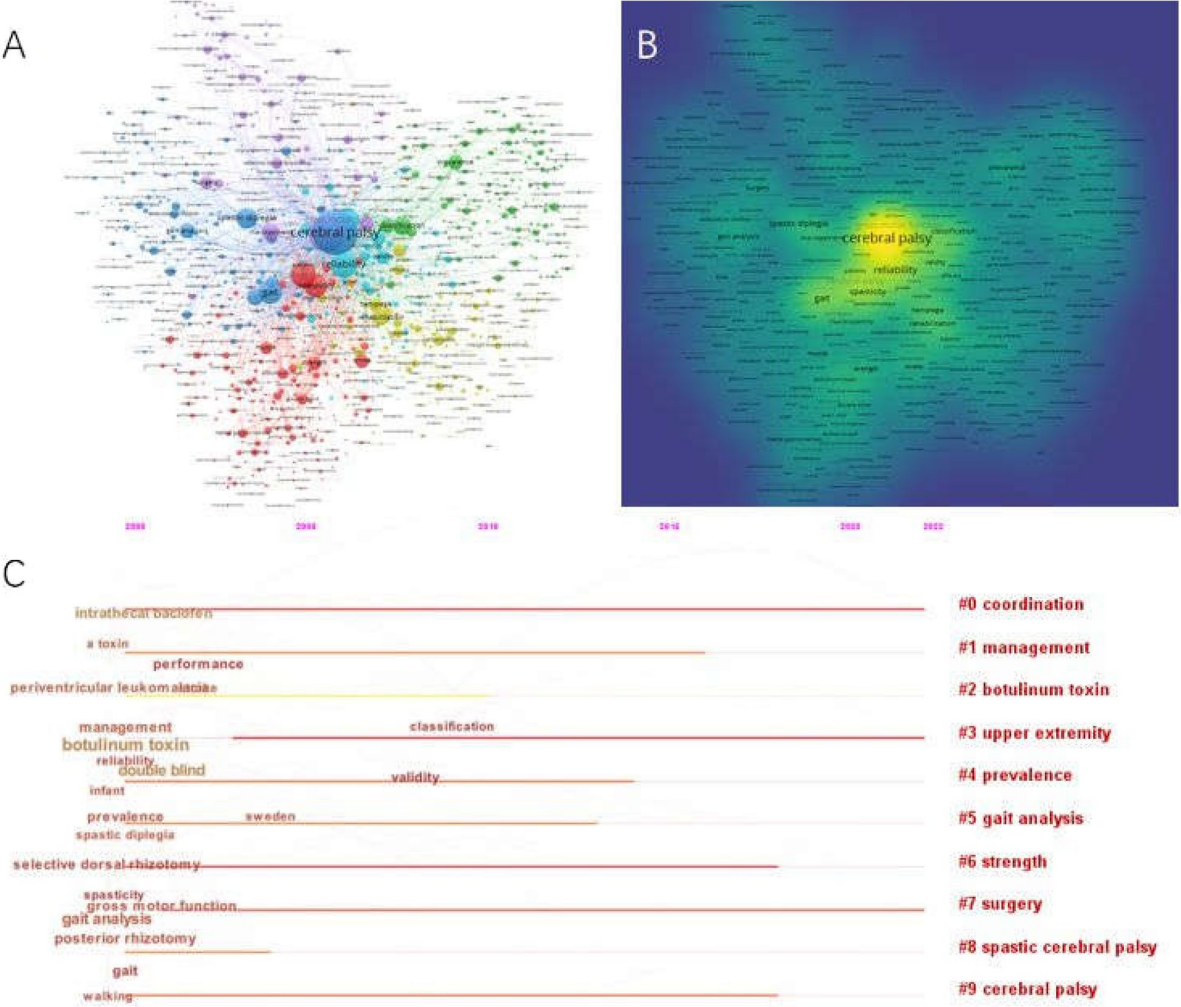
**A** The network of keywords generated by VOSviewe. **B** The density of keywords generated by VOSviewer. **C** The cluster of keywords generated by citespace

Table 6 shows the top 10 keywords (including Author Keywords and Keywords plus) co-occurrence frequencies. The word that came up most often was Cerebral palsy (2465), followed by children (1197), reliability (685), gross motor function (558), spastic diplegia (442), gait (349), management (313), classification (303), spastic cerebral palsy (286) and gait analysis (240). The top ten keyword clusters were selected for analysis and listed in Fig. 7. They were as follows: Coordination, Management, Botulinum toxin, Upper extremity, Prevalence, Gait analysis, Strength, Surgery, Spastic cerebral palsy, and Cerebral palsy.

**Table 6:** Top 10 keywords co-occurrence frequencies

	Keyword	Frequency	Centrality	Total link strength (TLS)
1	Cerebral palsy	2465	0.00	15,053
2	Children	1197	0.02	10,178
3	Reliability	685	0.06	5894
4	Gross motor function	558	0.13	4684
5	Spastic diplegia	442	0.09	3555
6	Gait	349	0.12	4572
7	Management	313	0.58	2415
8	Classification	303	0.04	2672
9	Spastic cerebral palsy	286	0.11	1678
10	Gait analysis	240	0.37	1961

### The burst of Keywords

**Fig. 8:**
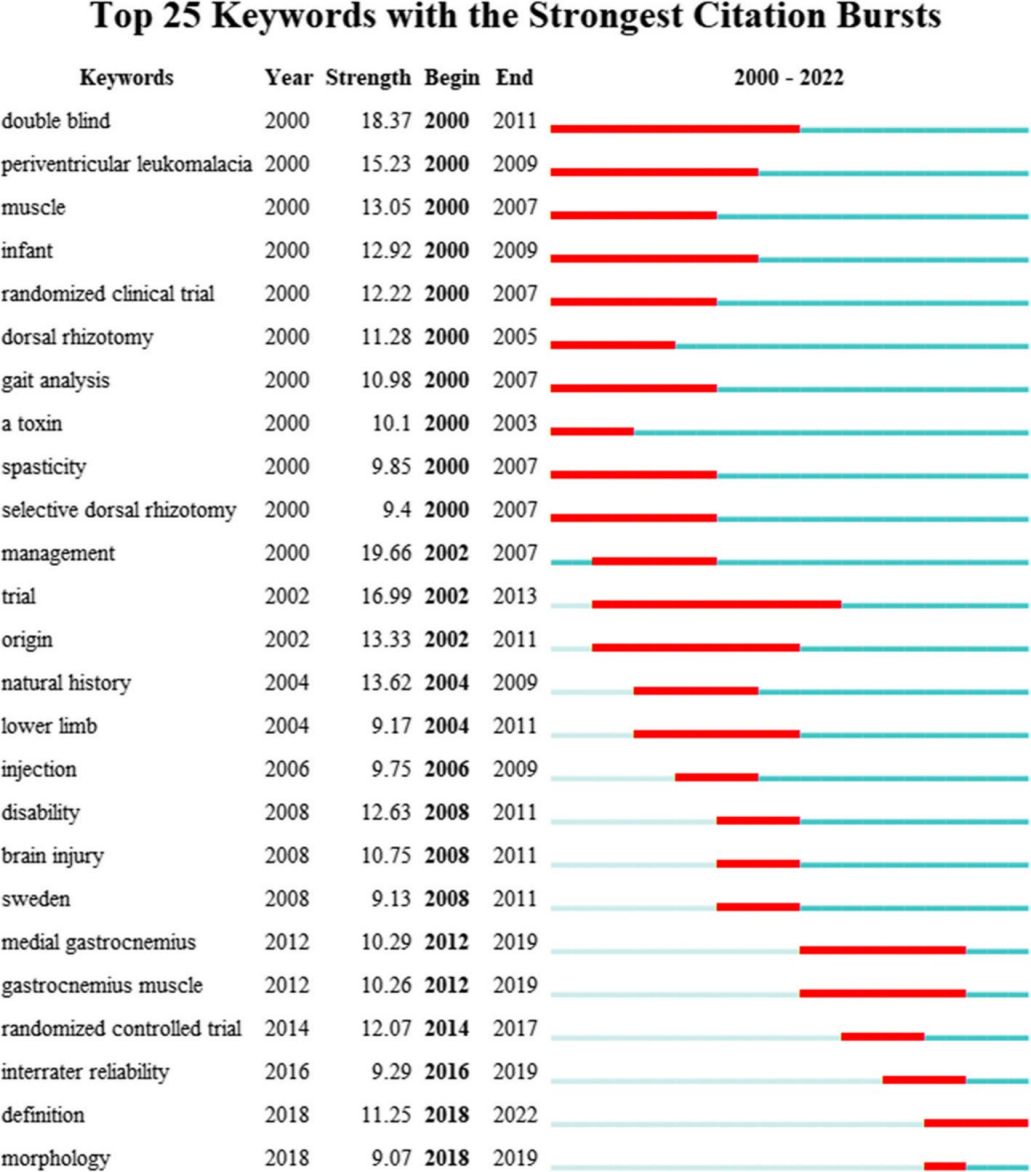
Top 25 keywords in terms of burst detection, where red bars indicate the burst year

Figure 8 shows the top 25 keywords in terms of burst detection. From 2000 to 2011, management, double blind, trial, periventricular leukomalacia, natural history, muscle, origin, and disability were commonly cited, with burst strengths reaching 19.66, 18.37, 16.99, 15.23, and 13.25, respectively. From 2012 to 2022, randomized controlled trial, definition, gastrocnemius muscle, interrater reliability, and morphology were commonly cited, with the burst strength reaching 12.07, 11.25, 10.29, 10.26, and 9.29 respectively.

## Discussion

To the best of our knowledge, this was the first bibliometric study on spastic CP to provide a comprehensive review of the developing trends of research in this field. According to the WoSCC database search, 3988 publications were identified from 1 January 2000 to 30 November 2022. The temporal and spatial distribution, author and institution contributions, and top journal were assessed by employing WoSCC literature analysis wire, VOSviewer, CiteSpace, and online analysis platform for bibliometrics. In addition, keyword analysis, cluster analysis, and burst hotspot analysis were used to identify the currently hot research areas and frontiers of research in spastic CP.

The publication trend can reflect the development speed and research progress. The number of studies related to spastic CP has steadily increased over the past twenty years, with approximately four times as many publications in 2020 as in 2000. This indicates that this field is a hot topic and will continue to attract increased attention in the future. Specifically, from 2000 to 2005, the relatively small amount of publications in this period indicates that the study of spastic CM is in its infancy. From 2006 to 2015, because a large number of researchers began to publish their study on spastic CP, the number of publications showed steady growth, then sharply increased after 2016.

According to the top countries/regions, institutions, authors, and journals in Table 1–4. The USA not only contributed the highest number of publications, total citation but also was the most cooperative country because spastic CP has been studied more intensively than in other countries, followed by the Netherlands, which was the second most publications and cooperation. In contrast, Australia had the largest number of citations per publication, which suggest that the USA, Netherlands, and Australia were the most influential countries in spastic CP. Western countries and regions have shown close cooperation, but A relatively low total link strength in South Korea has urged researchers to strengthen research further. Besides, in terms of institutions, nearly all of the top 10 institutions are from the top countries/regions with the most published papers, suggesting the good academic ability of the country in this field. Kathleen Univ Leuven and Vrije Univ Amsterdam were the most productive institution.

Regarding total citations and citations per publication, the highest is Royal Children’s Hosp. Desloovere K, who was the top productive author. Graham HK has the highest total citation and citations per publication. At the same time, Molenaers G has an advantage in cooperation. This indicates that these institutions and authors have tremendous influence in spastic CP fields, which can be selected for research cooperation. With respect to journals that published spastic CP studies, our data suggested that DEVELOPMENTAL MEDICINE AND CHILD NEUROLOGY had the largest number of publications, total citations, citations per publication, and total link strength. Although ARCHIVES OF PHYSICAL MEDICINE AND REHABILITATION was not in the top five largest number of publications, its co-citation was well.

Cluster analysis illustrates the direction of research hotspots in spastic CP, whereas burst detection explains the evolution of relevant hotspots over time. In this study, we combined the most cited references, top keywords, keywords clusters, and the burst of Keywords, the research frontiers, and hotspots of spastic CP were found to be as follows:

1. Definition of CP, epidemiological characteristics. Among the most-cited references, four explored these contents in spastic CP, especially there were two articles describing the changing panorama of CP in Sweden [32, 33]. Similar research has been carried out by Swedish scientists [34, 35]. The keyword of classification is the top eight words. The word of definition was a red bar from 2018 to 2020 in terms of burst detection, revealing that the topic still is a hotpot in this field.
2. Mechanisms of spastic CP. Chorioamnionitis and CP in term and near-term infants published in the PHYSICAL THERAPY and authored by Wu, YW in 2003, which pointed that risk factors of CP infants were as a possible role of chorioamnionitis [36]. Hagberg, H discussed ischemia–reperfusion and/or infection-inflammation were important factors of white matter damage (WMD) [37]. The intrinsic vulnerability of specific cell types and systems might be more critical in determining the final pattern of damage and functional disability [38]. The point of view was published in PEDIATRIC RESEARCH, authored by Johnston, MV, in 2001. Keywords of periventricular leukomalacia were red bar from 2000 to 2009 in burst detection, indicating that could be a reason for spastic CP.
3. Standard for clinical studies. Keywords of double blind randomized clinical trial and randomized controlled trial were highlighted in 2000–2001, 2000–2009, and 2014–2017, respectively, indicating that clinical trials had been a hotspot in the research of spastic CP for a long time. From the keywords clusters, we could note that many new treatments had been introduced into the treatment of spastic CP, such as botulinum toxin and surgery. De Beukelaer N assessed medial gastrocnemius (MG) growth in children by 3D-freehand ultrasound prior to and six months post-BoNT-A injections suggesting that re-injections should be postponed at least beyond half of a year [39]. Miyanji F studied the effect of surgery-related complications on clinical outcomes, concluding surgery in patients with CP leads to a significant improvement in health-related quality of life (HRQo) [40].
4. Spastic CP diagnosis and treatment management, efficacy evaluation, etc. the American Academy of Neurology and the Practice Committee of the Child Neurology Society reported the Practice parameter: Diagnostic assessment of the child with CP in NEUROLOGY [41]. Among the important keywords and clusters, management, classification, and management revealed the contents of diagnosis and treatment management. Gross motor function, interrater reliability, gait analysis and coordination revealed the contents of efficacy evaluation. More specifically, the efficacy evaluation included more details, such as the upper limbs and gastrocnemius muscles. Research of Elvrum AG pointed out that the Both Hands Assessment (BoHA) provides valid measures of hand use as suggested by its high correlation with other activity-based measures of hand function [42]. The research result of Boulard,C presented changing muscle and joint stiffness in children with CP as the effectiveness of interventions [43]. Trionfo A [44] introduced pain management(lumbar plexus nerve blocks, LPN) for children who underwent hip reconstruction and the results showed that LPN was an effective pathway.

There are inevitably several limitations to our study. First, the publications were only searched from the WoSCC database, which may lead to incomplete literature. Other databases such as Cochrane Library and Google Scholar. Scopus and PubMed may produce slightly different results. Nevertheless, the Web of Science database is the most popular and widely recognized database for bibliometric analyses. Second, we excluded non-English publications, which may lead to biased results. Finally, some bias during the selection of publications may not be avoided. For example, the same institution may have used different names at different periods, although two people were assigned to review the initial results.

## Conclusions

Research on spastic CP has grown rapidly, especially in the last five years. The USA, Netherlands, Australia, England, and other high-income countries contributed to the most publications in this area. Reliability, gross motor function, spastic diplegia, gait analysis, standardized management, clinical trial, and efficacy evaluation have been the most frequently studied spastic CP in recent years. A new treatment method, precise intervention, and reliable efficacy evaluation are helpful and a potential new direction in developing the spastic CP study.

## Data Availability

The original contributions presented in the study are included in the article. Further inquiries can be directed to the authors without undue reservation.
